# Performance on classroom simulations enhances preservice teachers' motivation in teaching: A latent change perspective

**DOI:** 10.1111/bjep.12761

**Published:** 2025-03-04

**Authors:** Hui Wang, Sophie Thompson‐Lee, Robert M. Klassen

**Affiliations:** ^1^ The Education University of Hong Kong Tai Po Hong Kong; ^2^ The University of Oxford Oxford UK

**Keywords:** career intentions, classroom simulation performance, motivation in teaching, person–vocation fit, preservice teachers, self‐efficacy

## Abstract

**Background:**

Preparing preservice teachers for teaching placements and future careers is crucial. However, their motivation often fluctuates as they gain experience and receive feedback from influential sources. While previous studies have examined changes in preservice teachers' motivation over time, there has been little research on how this motivation varies in relation to performance during simulations.

**Aims:**

We explored how performance on a series of classroom simulation sessions predicts preservice teachers' self‐efficacy, career intentions, and perceived fit with the profession, after controlling for the baseline levels.

**Sample:**

Participants were 1411 preservice teachers from an undergraduate teacher education programme in Australia (M = 20.27 years, SD = 4.54).

**Methods:**

Data were collected from students enrolled in an introduction to teaching course in a 4‐year teacher education programme. Participants completed three classroom simulation sessions spaced over a 3‐week period. We used latent change structural equation modelling to test the effects of performance on classroom simulations on preservice teachers' self‐efficacy, career intentions and perceived person–vocation fit.

**Results:**

The level of performance on classroom simulations significantly predicted changes in self‐efficacy and person–vocation fit (but not career intentions), even after controlling for baseline levels of the constructs, as well as gender and age. Moreover, the change in teaching self‐efficacy was progressively more pronounced after the second and third classroom simulation sessions. Finally, both age and gender were found to be associated with preservice teachers' motivation to teach.

**Conclusions:**

The implications for practice are that preservice teacher motivation may respond well to regular, repeated teaching‐related simulations.

## INTRODUCTION

It is crucial to adequately prepare preservice teachers for school placements, given the high stakes involved in the endeavour (Christophersen et al., 2016). A preservice teacher's motivation—seen in their effort, persistence and task choices—fluctuates throughout the teaching placement according to context‐related challenges (e.g. Rupp & Becker, 2021), but the motivation patterns set in school placements are associated with future success in the profession (Watt & Richardson, 2007). School placements offer an opportunity for professional growth and identity formation, but also pose very real risks for a range of stakeholders, including potential disruptions to pupils' learning progress due to a preservice teacher's inexperience, challenges for the receiving school in adapting to new staff and maintaining educational standards, and risks to the reputation of the initial teacher education (ITE) programme if preservice teachers struggle in the classroom (Flores, 2020). An additional risk is to the professional motivation and commitment of the preservice teachers themselves if they enter the school placement without adequate preparation. ITE programmes employ a variety of strategies to prepare preservice teachers for classroom entry, including comprehensive pedagogical training, experiential learning through role‐playing, and critical discussions on addressing potential classroom challenges (Darling‐Hammond & Bransford, 2005).

Research in recent years has suggested that preservice teachers' engagement in structured classroom simulation interventions (e.g. scenario‐based learning) could significantly increase their classroom readiness and teaching self‐efficacy (Ade‐Ojo et al., 2022; Klassen et al., 2023; McGarr, 2021). The current study implements classroom simulation activities for preservice teachers in Australia. It explores the relationship between their performance on these simulations and the resulting impact on their motivation to teach, including aspects, such as teaching self‐efficacy, career intentions and perceptions of person‐vocation fit (referred to as ‘PV fit’ in this article).

## PREPARING PRESERVICE TEACHERS FOR SCHOOL PLACEMENTS

### The importance of motivation in preservice teachers

The school placement, variously labelled as ‘field experience’, ‘teaching practicum’, and ‘clinical internship’, is a critical and consequential component of ITE. For many preservice teachers, the school placement is the first exposure to the challenges and rewards of classroom practice, with the experience serving as an important milestone on the path to establishing a sense of professional identity as a teacher (Anderson & Stillman, 2013). Specifically, preparing preservice teachers for their placements and fostering their motivation during these experiences are essential components of teacher education. Motivated preservice teachers are more likely to engage actively with their students and develop effective teaching practices that will subsequently improve student achievement (Richardson & Watt, 2006). In contrast, when preservice teachers lack motivation or confidence in their teaching abilities, they are at a greater risk of experiencing burnout and disengagement from the teaching profession (Skaalvik & Skaalvik, 2010). Therefore, teaching placements represent an important step in the transformation from student to teacher, and thus, careful preparation, often involving some form of approximation of teaching practice, is important to ensure success.

### Key motivational aspects

Among the various motivational factors considered important for preservice teachers, three key aspects have received considerable attention and have been extensively studied: teaching self‐efficacy, career intentions, and perceived PV fit (Klassen et al., 2023; Ma et al., 2022; Wang & Klassen, 2023; Wang et al., 2021). These three areas of motivation are closely related to each other but nevertheless represent three distinct aspects of preservice teachers' motivational lives. Specifically, Zee and Koomen (2016), as well as Wang et al. (2015) suggest that (preservice) teachers' beliefs in their teaching capabilities, that is, their teaching self‐efficacy, influence their persistence, resilience, and level of effort in teaching. Ma et al. (2022) further suggest that preservice teachers' career intentions or commitment reflect the extent to which they are motivated to pursue and then commit to a teaching career. Previous research has shown that preservice teachers with stronger career intentions are more likely to remain in the teaching profession for a longer period (Watt & Richardson, 2007). Finally, Klassen, Rushby, Durksen, & Bardach's (2021) PV fit framework, which is under the umbrella concept of person‐environment fit (Kristof, 1996), suggests that only when preservice teachers perceive a level of congruence between their values, skills, beliefs, and those associated with teaching, will they seriously consider and remain in the profession over the long term (see also De Cooman et al., 2019; Hayes & Stazyk, 2019). Fostering the motivation of preservice teachers—encompassing these three key aspects—requires a supportive learning environment. Providing support for preservice teachers' motivation is essential for shaping their long‐term engagement and success in the teaching profession (Richardson & Watt, 2010).

### Strengthening preservice Teachers' motivation through situated learning

Providing preservice teachers with a supportive learning environment involves more than merely building subject‐area knowledge and pedagogical skills; it is also the application of knowledge and skills in a *situated* context that defines success in placements (Levin, 2024; Mikeska et al., 2023). *Situated learning* was defined by Lave and Wenger (1991) as the process of learning that occurs in the context of social interactions and authentic activities, emphasizing that knowledge is constructed through participation in a community of practice. The development of situated learning can involve exposing preservice teachers to the complexity and subtle nuances inherent in the classroom context (Anderson & Stillman, 2013). Early‐stage teaching placements are notoriously demanding and stressful for preservice teachers (Ma & Cavanagh, 2018), and activities that promote contextualized, situated learning experiences can lead to more successful and adaptive motivation responses when preservice teachers enter the classroom for the first time (Brown et al., 2015). Providing preservice teachers with classroom experiences—simulated or otherwise—is a necessary step in developing situated learning and motivation responses necessary for successful classroom practice.

### Classroom simulations to build preservice Teachers' motivation via situated learning

Initial teacher education programmes frequently include various forms of simulated classroom experiences to help prepare preservice teachers for the classroom, including virtual or augmented reality (Huang et al., 2023), role‐playing (Moreno‐Guerrero et al., 2020), scenario‐based learning (Klassen et al., 2023), or vignette studies (Wang et al., 2024), all of which allow for exploration of approximations of practice associated with situated learning. The theories underpinning these simulations include situated learning theory (e.g. Lave & Wenger, 1991), pedagogical reasoning (Loughran, 2019) and Bandura's social cognitive theory (Bandura, 1997), each of which hypothesize that competence develops through the interaction of personal factors, environmental factors (i.e. the context), and behavioural factors (i.e. performance on various tasks). Simulation approaches have also been labelled as ‘case‐based learning’ or ‘near‐world simulations’ (Errington, 2011), with results from studies involving preservice teachers showing increases in confidence and classroom readiness (e.g., Klassen et al., 2023). From a motivational perspective, simulation tasks are closely aligned with the basic psychological need of competence outlined in self‐determination theory (Deci & Ryan, 2000). These tasks enhance preservice teachers' teaching self‐efficacy and preparedness for the classroom by offering opportunities for meaningful, hands‐on experiences in a classroom‐like setting. According to Bandura's framework, such mastery experiences are crucial for developing self‐efficacy, as they allow preservice teachers to practice and refine their skills in a supportive environment, which ultimately fosters a greater sense of competence and confidence in their teaching abilities (e.g. Hornstra et al., 2023). In a similar vein, participating in classroom simulations should enhance preservice teachers' perceived alignment between their attributes and skills and the demands of the teaching profession. This, in turn, should strengthen their career intentions in teaching and perceived PV fit (e.g. Klassen et al., 2023).

In real practice, classroom simulations have been found to be effective in promoting preservice teachers' motivation and teaching effectiveness. For example, in a recent study, Samuelsson et al. (2022) compared classroom simulations with avatar training and traditional training with peer support in seminars and found that the simulations were significantly more effective than peer training in promoting preservice teachers' self‐efficacy in teaching. Similarly, McPherson et al. (2011) utilized a web‐based simulated classroom in a special education context and found it significantly improved participants' perceptions of teacher preparedness and their attitudes towards inclusion in teaching practices. Additionally, Theelen et al. (2019) conducted a systematic review that concluded classroom simulations can enhance preservice teachers' classroom management, teaching skills and teaching self‐efficacy. Moreover, recent research on simulated classroom tasks has explored the effectiveness of scenario‐based learning (SBL) and interventions that include classroom simulations, space for reflection and feedback from experienced teachers. Results further showed that simulations by themselves are not sufficient to change motivation, but simulations accompanied by reflections and feedback make a significant difference in promoting motivation (Bardach et al., 2021).

### Linking simulation performance to motivation in teaching: Identifying the gap

Although previous studies on classroom simulations have specifically shown that the development of preservice teachers' motivation can be linked to their exposure to realistic classroom simulations (Gundel et al., 2019; Klassen et al., 2023; Samuelsson et al., 2022), the relationship between preservice teachers' actual performance on these simulations and changes in their teaching motivation remains unclear. It is possible that those who perform better on classroom simulations experience increased motivation due to a sense of accomplishment (Bandura, 1977; Tschannen‐Moran & Hoy, 2001). Conversely, those who perform well might already possess a high level of motivation, making it difficult to further enhance their motivation due to a ceiling effect (Koedel & Betts, 2010). Additionally, performance on classroom simulations may not be strongly linked to motivation, particularly in low‐stakes assessment environments, such as supplementary or optional learning sessions, where performance is typically not viewed as a critical reflection of a student's competence. In these contexts, participants' efforts may also be minimal (Attali, 2016; Wise & DeMars, 2005).

Therefore, a significant gap in research exists regarding the relationship between preservice teachers' actual performance on classroom simulations and their changes in motivation for teaching as we delve deeper into the role of classroom simulations in teacher education. Building this understanding is crucial, as preservice teachers' baseline motivations vary individually based on their accumulated life experiences (Rupp & Becker, 2021). Only by establishing a relationship between simulation performance and motivation can we ensure that classroom simulations are indeed effective. In this study, we are particularly interested in, first, how performance on classroom simulation sessions influences changes in preservice teachers' career‐related motivations, including self‐efficacy, career intentions, and perceptions of PV fit with teaching as a career, while controlling for baseline levels of motivation. Second, as classroom simulations may consist of either a single session (e.g. Samuelsson et al., 2022) or multiple sessions (e.g. Klassen et al., 2023), we aim to investigate whether participation in multiple simulation sessions, along with varying performance outcomes after each session, contributes to the cumulative development of motivation. To the best of our knowledge, this is the first study that has explored the links between changes in career‐related motivations and performance on classroom simulations. Our hypotheses were as follows.

Session‐specific performance on classroom simulation sessions will positively correlate with changes in career‐related motivations, including teaching self‐efficacy (H1a), career intentions (H1b), and perceived fit (H1c), after accounting for baseline values of these variables.

Repeated participation in a classroom simulation intervention will lead to progressive increases in teaching self‐efficacy (H2a), career intentions (H2b) and perceived fit over time (H2c). To assess this hypothesis, we expect the effect sizes—or the strengths of the relationships—between simulation performance and preservice teachers' changes in motivation to be stronger in later sessions compared with earlier ones.

## METHOD

### Development of classroom simulations

The classroom simulation intervention was made up of three 1‐h online sessions delivered over a 3‐week period of one semester. Each session included five short video and/or text scenarios resulting in participants seeing a total of 15 scenarios based in early years, primary, or secondary education. The scenarios depicted authentic situations one would encounter as a teacher, for example, working with parents, managing disruptive students and classroom transitions. Although participants represented different teaching levels (e.g. primary, secondary, early years), we chose scenarios that reflected general classroom challenges that were relevant for all teaching levels.

The content of the scenarios was created using a *critical incidents* approach from over 20 individual interviews and focus groups conducted with practicing teachers with more than 5 years' teaching experience. The experienced teachers were asked to identify challenges that preservice teachers might expect to face in a school placement. Scenarios were iteratively developed by writing and revising the content with a team of writers, including experienced teachers. Next, we created animated videos based on the scripts, with the animated classroom environment and characters vetted by an additional panel of experienced teachers who gave advice on the authenticity of the videos. We then created a scoring key through administering the scenarios to a ‘concordance panel’ of 20 experienced teachers who provided suggested responses to the scenarios (e.g. *Rate the appropriateness of this response…*) The possible choices included ‘very appropriate’, ‘somewhat appropriate’, ‘somewhat inappropriate’, and ‘very inappropriate’. Any disagreements on appropriate courses of action were resolved through group discussion, and in some cases, by dropping scenarios with scoring differences that were not easily resolved. Post‐scenario participant feedback (e.g. *You could also consider pairing the student with a more able* peer) was created over the period of 1 year through a series of workshops with experienced teachers and teacher educators from the UK and Australia, with a consensus approach used to determine final feedback messages. Feedback messages were automatically tailored based on the congruence of participants' scoring responses with expert responses.

### Procedures

The simulated classroom sessions were completed online using the participants' choice of digital device. The sessions were completed over 3 weeks with a 1‐week interval between each session. Each session lasted for around 30–60 min and was made up of five simulated classroom experiences. Data were collected in 2020 and 2021 when classroom placements were interrupted by the pandemic.

Before the simulation sessions, participants completed a pre‐test questionnaire. This consisted of questions about demographic information and measures of self‐efficacy, PV fit and career intentions. After each session, a post‐test questionnaire was administered, re‐assessing self‐efficacy, PV fit and career intentions. At the end of each session, participants were given a report indicating how closely their responses matched those of experienced teachers. They were also given automatically generated (i.e. undifferentiated teaching suggestions for a particular classroom scenario) ideas about how to improve their decision making in the future sessions. In total, four survey responses were collected, including one pre‐test and three post‐test surveys.

After reading or watching the scenario, preservice teachers were presented with three possible responses to the scenario, and they were then guided to use a 4‐point scale to rate the appropriateness of each possible response (e.g. *Rate the appropriateness of the following responses to the scenario*), from ‘very inappropriate’, ‘inappropriate’, ‘appropriate’, to ‘very appropriate’. Scoring alignment (between participants and the key established by experienced teachers) was calculated as follows. For each response option, the maximum score was three points if the participant's rating was the same as the optimum rating as deemed correct by a panel of expert teachers. For example, if the participant rated option 1 as ‘somewhat appropriate’ in line with the scoring consensus (i.e., set by a panel of experienced teachers), then the participant would score three points. The score declined (i.e., 2, 1, 0) as the distance from the experienced teacher rating increased (i.e. one place away from the expert consensus earned 2 points; two places away earned 1 point, etc.). The total possible score for each session was 45 (3 points per response option × 3 response options × 5 scenarios). To facilitate easier interpretation and comparison of scores, we have then converted all scores to a 100‐point scale.

### Participants

Participants were 1411 preservice teachers, aged between 18 and 40 years old (M = 20.37, SD = 4.54), from an undergraduate teacher education programme in eastern Australia. Students in this programme complete 4 years of academic study that includes academic discipline studies coupled with studies in educational theory, curriculum and pedagogy, all interspersed with professional experience placements. Most of the participants were female (75%) and self‐identified as ‘white European’ or ‘Australian’ (94%) and in the first year of their 4‐year programme (89.2%). Most participants were studying to be teachers in primary schools (56.5%), followed by secondary schools (35.8%) and early years settings (7.7%).

A small portion of the data was found to be missing. More specifically, Session 1 had 0.1% missing data, Session 2 had 3.6% missing data, and Session 3 had 6.2% missing data. The analysis of the missing data indicates that it was *missing completely at random*. This is supported by the non‐significant result of Little's MCAR test [*χ* ^2^(41) = 52.35, *p* = .110].

### Measures

#### Teachers' self‐efficacy

Three items adapted from the Teachers' Sense of Efficacy Scale (Tschannen‐Moran & Hoy, 2001) were used to measure self‐efficacy for teaching before and after the intervention. These items have been previously measured for reliability and validity (Klassen, Rushby, Maxwell, et al., 2021; Wang et al., 2024). The three items used a 6‐point Likert scale (1 = *strongly disagree*, 2 = *disagree*, 3 = *somewhat disagree*, 4 = *somewhat agree*, 5 = *agree*, 6 = *strongly agree*). The items were as follows:

- I am confident that I can manage classroom behaviour.
- I am confident that I can develop effective teaching strategies.
- I am confident that I can help all students value learning.

#### Career intentions

Intentions for pursuing a teaching career were measured with three items adapted from Hackett et al.'s (2001) Occupational Commitment scale. Previous use of this measure shows adequate reliability and evidence of construct validity (Klassen et al., 2023). The three‐item measure used a 6‐point Likert scale ranging from 1 = *strongly disagree* to 6 = *strongly agree*. The three items were as follows:

- I am enthusiastic about teaching as a career.
- Teaching is a likely profession for me.
- I am excited to train as a teacher.

#### Person‐vocation fit

PV fit was measured using a three‐item, 6‐point Likert scale adapted from Chuang et al. (2016). The three‐item measure used a 6‐point Likert scale with scores ranging from 1 = strongly disagree to 6 = strongly agree. Previous use of this brief measure shows adequate reliability and evidence of construct validity (Chuang et al., 2016; Klassen et al., 2023). The three items were:

- There is a close match between my skills, knowledge, and abilities and those required for a teaching career.
- There is a close match between my personal characteristics (e.g. personality) and those required for a teaching career.
- There is a close match between my interests and those required for a teaching career.

### Rationale for analyses

#### Preliminary analyses

Preliminary analyses included descriptive statistics and zero‐order correlations across multiple sessions of the classroom simulation. Confirmatory factor analyses were also employed to assess the construct validity of the study measures. To determine the goodness‐of‐fit, several indices were considered, including the Comparative Fit Index (CFI), Tucker–Lewis Index (TLI), root mean square error of approximation (RMSEA), and standardized root mean square residual (SRMR). According to established criteria (e.g. Hu & Bentler, 1999; Kline, 2018), RMSEA and SRMR values below 0.06 and 0.08, respectively, along with CFI/TLI values greater than 0.95 and 0.90, indicate excellent and acceptable fit to the data.

#### Hypothesis testing using latent change structural equation modelling

To examine the impact of participants' performance on the classroom simulation sessions on changes in their teaching self‐efficacy, career intentions, and perceived PV fit over the entire study period, a latent change structural equation modelling analysis was implemented. This analysis aimed to explore the relationships between the three scores representing participants' performance on the simulation sessions and the changes observed in their teaching self‐efficacy, career intentions, and PV fit from the pre‐session to the end of Session 3. More specifically, the paths from the three simulations' scores to the changes in participants' teaching self‐efficacy, career intentions, and PV fit were modelled. These paths aimed to highlight the longitudinal connections, examining whether the performance on each session was associated with any changes in the outcome variables across the three simulation sessions, while accounting for the baseline values of these variables.

Additionally, the model was constructed by assigning loadings of 1 to the outcome variables (teaching self‐efficacy, career intentions, and perceived fit) at all four data collection points (pre‐session, Sessions 1–3), forming the latent intercept factor (baseline). Loadings ranging from 0 to 3 were set for the latent change factors corresponding to the pre‐session (0), Session 1 (1), Session 2 (2), and Session 3 (3) respectively. The latent change constructs, therefore, represent the overall patterns of change in preservice teachers' motivation across all three simulation sessions. The intercepts for all observed items were fixed at 0. To specifically test the study hypotheses, we regressed the latent intercepts and latent changes in preservice teachers' self‐efficacy, career intentions, and perceived PV fit on their performance in each of the classroom simulations.

The performance of each session was represented by a single score, which combined the participants' performance across five different classroom scenarios in each session. All outcome variables were assessed as latent variables, with each variable being informed by three indicators. Model parameters were estimated using a robust maximum likelihood (MLR) estimator, a variation of the maximum likelihood (ML) estimator developed by Muthén and Muthén (1998‐2017). To effectively handle missing data, the model parameters were estimated using *Full Information Maximum Likelihood* (FIML) in M*plus* (Muthén & Muthén, 1998‐2017), following recommendations by Enders and Bandalos (2001) and Schlomer et al. (2010). Correlations were modelled between the same items across multiple time points of data collection. Finally, gender and age were included as covariates.

## RESULTS

### Preliminary analyses

The comprehensive confirmatory factor analysis (CFA) conducted on all study variables across the three sessions revealed excellent fit indices (CFI = .983, TLI = .977, RMSEA = .027, SRMR = .035) with factor loadings ranging from .667 to .918. Descriptive statistics and zero‐order correlations can be found in Tables 1 and 2. Notably, the correlations among participants' motivational constructs across all four data collection points were significant at *p* < .05. However, relatively weaker correlations were observed between simulation performance and the motivational constructs. Specifically, participants who performed better in Session 1 tended to have lower levels of self‐efficacy in the pre‐test but higher levels of self‐efficacy after Session 1. They also reported a higher PV fit following Session 1. Furthermore, participants who performed better in Session 2 exhibited greater self‐efficacy after all three sessions (three post‐tests), increased career intentions after Sessions 2 and 3, and higher PV fit across all four data collection points (the pre‐test and the three post‐tests). Finally, participants who performed better in Session 3 displayed higher levels of self‐efficacy after Session 3, as well as increased career intentions in the pre‐test and after Sessions 2 and 3, along with higher PV fit following Sessions 2 and 3.

**TABLE 1:** Descriptive statistics of study variables.

Variables	M				SD				*α*			
	Pre‐session	S1	S2	S3	Pre‐session	S1	S2	S3	Pre‐session	S1	S2	S3
Simulation performance	—	83.49	78.23	76.97	—	6.53	5.87	6.23	—	—	—	—
Self‐efficacy	4.82	5.18	5.07	5.07	0.72	0.65	0.66	0.72	.782	.803	.813	.862
Career intentions	5.47	5.56	5.52	5.48	0.67	0.63	0.66	0.70	.873	.897	.908	.932
Person‐vocation fit	5.14	5.34	5.33	5.34	0.65	0.63	0.62	0.67	.773	.831	.835	.890

*Note*: S1 = session 1; S2 = session 2; S3 = session 3.

**TABLE 2:** Zero‐order correlations among study variables across sessions.

Variables	1	2	3	4	5	6	7	8	9	10	11	12	13	14
1. Simulation performance (S1)	—													
2. Simulation performance (S2)	.104**	—												
3. Simulation performance (S3)	.172**	.051	—											
4. Self‐efficacy (pre‐session)	−.075**	.036	−.012	—										
5. Self‐efficacy (S1)	.099**	.065*	.009	.696**	—									
6. Self‐efficacy (S2)	−.027	.142**	.010	.539**	.593**	—								
7. Self‐efficacy (S3)	−.005	.064*	.138**	.492**	.529**	.694**	—							
8. Career intentions (pre‐session)	.011	.021	.061*	.387**	.393**	.299**	.321**	—						
9. Career intentions (S1)	.047	.034	.046	.354**	.499**	.357**	.346**	.839**	—					
10. Career intentions (S2)	.009	.061*	.058*	.247**	.317**	.460**	.418**	.663**	.708**	—				
11. Career intentions (S3)	.002	.056*	.083**	.259**	.340**	.397**	.535**	.620**	.655**	.754**	—			
12. Person‐vocation fit (pre‐session)	.001	.062*	.037	.495**	.465**	.379**	.339**	.571**	.534**	.394**	.371**	—		
13. Person‐vocation fit (S1)	.087**	.077**	.037	.437**	.619**	.451**	.413**	.489**	.605**	.420**	.420**	.725**	—	
14. Person‐vocation fit (S2)	.028	.120**	.084**	.359**	.419**	.577**	.501**	.445**	.497**	.631**	.557**	.563**	.600**	—
15. Person‐vocation fit (S3)	.018	.102**	.107**	.328**	.394**	.484**	.632**	.429**	.474**	.541**	.704**	.485**	.536**	.677**

*Note*: S1 = session 1; S2 = session 2; S3 = session 3.

* *p* < .05.

** *p* < .01.

### Latent change structural equation modelling

To further explore the relationship between performance on the simulation sessions and changes in participants' outcomes over time, a latent change analysis was conducted. Directional paths were modelled from each simulation score to the changes in the three outcome variables, while controlling for the baseline levels of these variables. This analysis provided a robust examination of the impact of the simulation sessions on participants' increases in teaching self‐efficacy, career intentions, and perceived PV fit.

**FIGURE 1:**
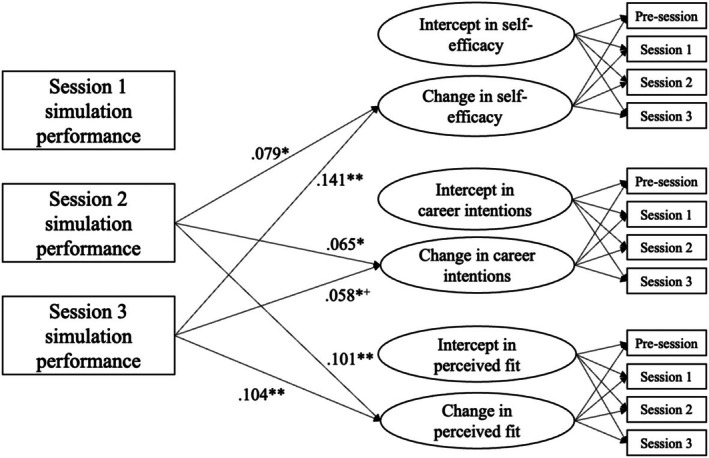
Results of the latent change analyses. *p < .05. **p < .01. Age and gender were used as covariates in this model. Paths from simulation scores to the intercepts of self‐efficacy, career intentions, and perceived fit were not modelled. They were simply presented in the model to help create the change constructs. Only the significant paths are presented in the figure. +indicates marginal significance.

As depicted in Figure 1, the results showed that participants' simulation performance in Session 1 did not significantly influence any changes in the outcome variables. However, in Session 2, higher performance in the simulations was significantly associated with an overall positive change (increase) in participants' teaching self‐efficacy (*β* = .079, *p* = .014), career intentions (*β* = .065, *p* = .039), and perceived PV fit (*β* = .101, *p* = .002) during the classroom simulations. In Session 3, higher performance had a stronger impact compared with Session 2 on the change in participants' self‐efficacy (*β* = .141, *p* < .001) and perceived fit (*β* = .104, *p* = .001), but not on career intentions (*β* = .058, *p* = .055).

Furthermore, covariate analyses revealed that participants who were older tended to have lower baseline levels of self‐efficacy compared with younger participants (*β* = −.074, *p* = .014), but they performed better in Session 1 (*β* = .074, *p* = .007; but not in the other sessions). Gender was also found to be a significant factor. Female participants exhibited stronger baseline career intentions (*β* = .078, *p* = .008) and perceived fit in the teaching profession (*β* = .083, *p* = .004) compared with their male counterparts, and they also tended to perform better in Session 1 (*β* = .070, *p* = .012; but not in the other sessions). Evaluating the entire model, participation in three simulation sessions significantly predicted 3% of the variability in participants'– increases in teaching self‐efficacy (*p* = .013) and 3% of the variability in their increases in PV fit (*p* = .008). However, it accounted for only 1% of the variability in changes in career intentions, which were not statistically significant (*p* = .128).

## DISCUSSION

In this study, we wanted to test how preservice teachers' performance on three classroom simulation sessions influenced changes in three motivation‐related outcome variables: teaching self‐efficacy, career intentions and perceptions of fit with the profession. Our design with multiple simulation sessions meant that we could test effects over time while controlling for baseline scores. It is worth noting that throughout the sessions, we have observed a consistent decrease in participants' performance. Given the diversity of tasks across sessions, the observed decline may have several possible interpretations. For example, the tasks may become more difficult (although not the researchers' original intention), making it less likely for participants to perform better. It is also possible that as participants became more familiar with the intervention, they may complete the questions more quickly, which could also lead to carelessness and a subsequent decrease in performance.

Findings from latent change SEM suggest that scoring levels from Session 1 were not associated with outcome variables, but in Session 2, higher scores were significantly associated with increases in self‐efficacy, career intentions and PV fit. Session 3 performance had a significant impact on self‐efficacy and fit but not career intentions. Although Sessions 2 and 3 tended to become more effective and started to bring significant improvements in participants' motivation in teaching, analyses across all three sessions indicated that the overall classroom simulation could bring significant improvement to preservice teachers' self‐efficacy and PV fit but not career intentions. Therefore, our Hypothesis 1a and 1c were supported.

Regarding the progressive increases in preservice teachers' motivation after each session, only the aspect related to teaching self‐efficacy was supported (H2a, but not H2b or H2c). The influence of simulation performance on changes in participants' self‐efficacy was stronger in Session 3 (.141) than in Session 2 (.079). For PV fit, performance in both Session 2 and Session 3 similarly predicted its changes. In contrast, the predictive power for career intentions decreased from .65 (which was statistically significant) to .58 (which became non‐significant) from Session 2 to Session 3. These results support our initial assertion that these three motivational aspects are closely related yet represent distinct dimensions of preservice teachers' motivational experiences.

Specifically, regarding teaching self‐efficacy, the results showed that the level of performance on the simulation sessions was associated with changes in teaching self‐efficacy (after exposure to > one session). This finding is predicted through Bandura's self‐efficacy theory, where successful, accumulated, and enactive experience is a key source of growth in perceptions of personal capabilities (Bandura, 1997). Our participants were at the very beginning of their teaching journey, although almost all of them will have had classroom experience in their role as students; most would have had limited exposure to realistic classroom situations where they took on the role of the teacher. This lack of experience may explain the finding that performance in Session 1 had little influence on changes in self‐efficacy. With increasing exposure to the intervention, that is, in Sessions 2 and 3, participants were more likely to experience increases in their teaching confidence. Therefore, to promote increases in teaching self‐efficacy, the provision of multiple simulation sessions is essential (Desimone, 2009; Kraft et al., 2018).

Regarding PV fit, we found that performance on a single classroom simulation session had no significant effect on changes in perceptions of fit, but the effects of exposure accumulated with repeated exposure: performance on Sessions 2 and 3 was associated with changes in perceptions of perceived fit. In this context, our results suggest that two simulation sessions—despite differing content, with the first likely serving as an initiation and the second as reinforcement—may be sufficient to promote changes in preservice teachers' perceived PV fit.

Furthermore, the pattern of change in career intentions was more nuanced: the first session resulted in little change, the second session was significantly associated with change, and the third session again showed a non‐significant change in career intentions. It may be that repeated exposures to classroom simulations do not change attitudes about career intentions: the commitment to the profession of preservice teachers may be harder to shift once established and realistically may be formed through a range of external sources. For example, Furlong (2013) and Hong (2010) both examined how (preservice) teachers' professional commitment and career intentions could be strongly shaped by societal narratives, media portrayals and social expectations. Simulations that emphasize skill development and practical application may not be sufficient to change preservice teachers' perceptions of the profession. While they can provide valuable experiences, these positive outcomes may not effectively counterbalance the preconceived doubts and concerns that preservice teachers have about teaching, such as perceptions of low status, inadequate support and limited opportunities for career advancement (Keller‐Schneider et al., 2020; Troesch & Bauer, 2020).

Finally, we have also explored the effects of demographic variables (age and gender) on longitudinal relationships between performance on the simulations and changes in outcomes. We found few meaningful differences, but older participants had slightly lower levels of self‐efficacy than younger participants at baseline, coupled with slightly higher performance in the first session. Past research has shown that teachers' self‐efficacy fluctuates with age (Klassen & Chiu, 2010), but with age comes self‐awareness: older participants may have been less prone to inflated self‐efficacy beliefs and more likely to accurately calibrate their self‐efficacy with actual performance. Gender differences were also modest, with higher baseline career intentions and fit with the profession for female participants, which is consistent with findings from Giersch (2021), indicating that among U.S. teachers, female students considering a career in teaching were more likely to be intrinsically motivated, whereas their male counterparts tended to be more extrinsically motivated.

### Practical implications of the findings

The results raise new questions about interventions designed to prepare preservice teachers for the classroom, and more specifically about the nature and pattern of online interventions for preservice teachers. We do not know very much about the optimal level of intensity of interventions when preparing pre‐professional teachers: how much exposure to an intervention is enough to bring about change? How many sessions are too many? We developed this intervention based on previous research and theory (e.g. research on teacher self‐efficacy and situated learning) to be authentic, scalable and effective in boosting preservice teachers' motivation for teaching. However, we did not have much guidance from past research on how to optimize the balance between number of sessions and effectiveness of the intervention. The findings from this study do not offer definitive findings about the optimal intensity of interventions, but there are hints that the effects of the classroom simulations accumulated over time: One session alone was not associated with a significant change in our outcome variables, which did not support Samuelsson et al.'s (2022) recent one‐shot simulation training that promoted preservice teachers' teaching self‐efficacy. Our findings further suggested that two sessions showed better results, particularly for promoting self‐efficacy and perceived fit within the teaching profession. While three sessions did not lead to notable changes in perceived fit, they still demonstrated a further progressive improvement in teaching self‐efficacy.

It is, therefore, possible that the skills and knowledge acquired in Session 1 laid the groundwork for Sessions 2 and 3, but Session 1 itself may have had only limited (and non‐significant) effects on the overall increase in motivation. The changes observed in the subsequent sessions could be influenced by the prior learning from Session 1. This is particularly relevant in this study, given that the content across the three sessions consists of 15 different scenarios, requiring participants to complete a certain number of tasks for their motivation to be significantly promoted. However, even with these new insights into the intensity of exposure to classroom simulations for preservice teachers, considerably more work is needed to understand how to bring about lasting change; our study does not offer very much insight into patterns of longer‐term change, and whether the changes we observed were maintained beyond a relatively brief period.

### Limitations

Several limitations of the study need to be considered when interpreting the results. First, due to the nature of the teacher education programme's ‘hurdle’ requirement for this intervention, we did not have a control group that did not participate in the intervention in the same setting. Similarly, we were not able to control the day‐to‐day learning taking place from instruction and experiences associated with the teacher education programme, and changes in the outcome variables were potentially influenced by a host of factors external to our intervention, including pandemic‐related factors that we did not measure (López‐García et al., 2022). However, ratings of the three outcome variables took place within the intervention context, and it is probable that participants connected the post‐test measures to the immediately preceding intervention. Moreover, since we only administered three cycles of the intervention, it is possible that the ‘sweet spot’ for the number of this intervention could be a number greater than three. For example, in a recent study on scenario‐based learning using five measurement points (one pre‐test and four post‐tests), we observed an ‘inverted U‐shaped’ (i.e. non‐linear) response pattern of teaching‐related career intentions (Klassen et al., 2023). Furthermore, it should also be noted that the individual elements of the intervention (i.e. scenarios, reflection, feedback) were not evaluated individually, and thus we do not know the relative importance of each element. Finally, we did not include a longer‐term follow‐up measurement, which could have shed light on the enduring effectiveness of the intervention; a longer‐term follow‐up measurement is clearly indicated in future research.

### Conclusions

In this study, we found that preservice teachers' performance on classroom simulations was associated with their confidence to teach (teaching self‐efficacy) and their perceptions of fitting in with the demands of the profession (PV fit). Our study's results supported the idea that online and scalable simulation‐based interventions could provide an easily administered and scalable supplement to conventional teacher education preparations, and importantly, could significantly increase the likelihood that preservice teachers enter the teaching placement with more confidence and a better perceived alignment with the teaching profession. Given that attrition rates among early‐career teachers are at an all‐time high (e.g. Walker, 2023) and promoting teacher self‐efficacy remains a persistent challenge across various instructional contexts (e.g. Mok et al., 2023; Täschner et al., 2024), authentic practice‐based interventions that are consistently linked to increased confidence, commitment, and a sense of fit are worth exploring and implementing.

## AUTHOR CONTRIBUTIONS

**Hui Wang:** Conceptualization; methodology; formal analysis; writing – original draft; writing – review & editing. **Sophie Thompson‐Lee:** Conceptualization; methodology; writing – original draft; writing – review & editing. **Robert M. Klassen:** Conceptualization; project administration; funding acquisition; supervision; writing – original draft; writing – review & editing.

## Data Availability

The data that support the findings of this study are available from the corresponding author upon reasonable request.
